# Safety and Effectiveness of Coronary Sinus Reducer in the Therapy of Refractory Angina Pectoris—Mid-Term Results of the Real-Life Cohort

**DOI:** 10.3390/jcm13154413

**Published:** 2024-07-28

**Authors:** Szymon Włodarczak, Piotr Rola, Artur Jastrzębski, Karol Turkiewicz, Andrzej Korda, Piotr Włodarczak, Mateusz Barycki, Jan Jakub Kulczycki, Łukasz Furtan, Adrian Włodarczak, Maciej Lesiak

**Affiliations:** 1 Department of Cardiology, Copper Health Centre (MCZ), 59-300 Lubin, Poland; a.jastrzebski@mcz.pl (A.J.); karolt777@gmail.com (K.T.); andkorda@wp.pl (A.K.); piotrwlodrczak123@gmail.com (P.W.); jan.jakub.kulczycki@gmail.com (J.J.K.); wlodarczak.adrian@gmail.com (A.W.); 2 Department of Cardiology, Provincial Specialized Hospital, 59-200 Legnica, Poland; piotr.rola@gmail.com (P.R.); mateusz.barycki@gmail.com (M.B.); lukas.furtan@gmail.com (Ł.F.); 3 1st Department of Cardiology, University of Medical Sciences, 61-701 Poznan, Poland; maciej.lesiak@skpp.edu.pl

**Keywords:** refractory angina pectoris (RA), coronary artery disease (CAD), percutaneous coronary intervention (PCI), coronary artery bypass grafting (CABG), coronary sinus reducer (CSR), quality of life (QoL)

## Abstract

**Background:** Despite continuous improvements in revascularization techniques, refractory angina without potential revascularization options remains a relevant clinical issue with significant impact on the patient’s quality of life. Recently, a novel device, the Coronary Sinus Reducer (CSR), has been introduced into clinical practice as a therapeutic option for patients with disabling angina pectoris. In this single-center, observational study, we evaluated the mid-term (3-month) safety and efficacy of the CSR in a real-world cohort. **Methods:** The study population consisted of 55 patients with refractory angina without potential revascularization options, who were predominantly men (87.3%) with a high cardiovascular risk factor burden and advanced angina (baseline CCS angina class 3.15 ± 0.6). In terms of procedure safety, all patients underwent successful device deployment with only one periprocedural complication. **Results:** At the 3-month follow-up, we observed a statistically significant improvement in angina control measured CCS class and SAQ-7 total questionnaire along with increased abolition of physical limitation—6-MWT (233.3 ± 107.1 vs. 305.2 ± 126.8; *p* < 0.0001). Additionally, we observed significant improvement in terms of quality of life measurements SF-36, the EQ-5D-5L questionnaire, and the EQ-VAS. **Conclusions:** Our real-world data suggest that CSR implantation is a relatively safe procedure and appears to be particularly effective in relieving angina symptoms and improving quality of life in subjects with refractory angina.

## 1. Introduction

Despite undeniable improvements in the conservative and interventional management of coronary artery disease, clinicians are still faced with the daily management of patients with refractory angina (RA).

Refractory angina is defined as persistent symptoms (>3 months) due to established reversible ischemia that is not adequately controlled with optimal medical therapy and for which appropriate revascularization options are not available. The proportion of patients with RA is increasing, ranging from 2% to even up to 20% [1,2], and unfortunately, there are no adequate therapeutic options.

The vast majority of cases of RA is associated with patients with advanced, diffuse CAD, yet the pathophysiology of RA is far more heterogeneous and involves coronary disease other than obstructive CAD [3]. Patients with RA have significant morbidity and quality of life impairments. In addition, their treatment results in significantly increased healthcare costs [4].

Several therapeutic agents aimed at improving myocardial perfusion (beyond traditional revascularization or anti-ischemic drugs) have been developed to overcome the current limitations of contemporary clinical practice in the management of the RA subpopulation [4]. Since 2007 [5], when the first-in-man study of the Coronary Sinus Reducer (CSR) device was conducted, we can observe a slowly growing body of scientific evidence supporting the use of the CSR in RA patients. The Neovasc Reducer (Neovasc Inc., Richmond, VA, Canada) is a percutaneous, balloon-expandable, hourglass-shaped stainless steel stent that creates a focal narrowing of the CS and increases coronary venous pressure. It is postulated that the increased venous backward pressure in the cardiac microcirculation promotes blood redistribution, improves the endocardial/epicardial blood flow ratio, and alleviates the symptoms of angina [6,7].

In this single-center prospective observational study, we evaluated the mid-term safety and efficiency of CSR in a “real-life” cohort with RA.

## 2. Materials and Methods

### 2.1. Study Population

The study population consisted of all consecutive patients who underwent the procedure of implantation of the Coronary Sinus Reducer in the Department of Cardiology of the Copper Health Center in Lubin, Poland, between May 2022 and January 2024. All subjects enrolled in the study had a primary diagnosis of chronic refractory angina (Canadian Cardiovascular Society (CCS) classes 2–4) for at least 3 months before the procedure in spite of maximum tolerable medical therapy for angina (at least 3 different drug groups). Only individuals without a recent history of acute coronary syndrome or recent coronary revascularization were included in the study (the blanking period for the recruitment process was 3 months). All patients prior to the procedure underwent evaluation by the local heart team for potential revascularization options. In addition, patients with chronic heart failure (New York Heart Association (NYHA) class 3–4), severe left ventricular systolic impairment (less than <25%), or high mean right atrial pressure (greater than 15 mm Hg) were excluded from the study.

All subjects signed a written consent for the procedure and participation in the study. The study was also approved by the local ethics committee (Bioethics Committee of the Lower Silesian Medical Board in Wroclaw—approval number 02/BOBD/2022)). In addition, the study was registered and approved by clinicaltrials.gov (NCT06288165).

### 2.2. Coronary Sinus Reducer Device and the Implantation Procedure

The Coronary Sinus Reducer (Neovasc Inc., Richmond, VA, Canada) is an hourglass-shaped stainless steel device mounted on an expendable balloon-catheter delivery system that operates in a standard “over-the-wire” fashion way. The delivery system has three radiopaque markers, two of which are attached to the distal edges of a scaffold, while the third additional marker is attached proximal to the balloon and is used to determine the exact position of the device during the implantation procedure.

The procedure is performed by vascular access located in the right Jugular vein (in selected cases, it is possible to perform the procedure through the femoral approach). Under local anesthesia, an introducer sheath (9 Fr) is inserted into the vein system. After the advancement of the multipurpose diagnostic catheter into the right atrium by the standard 0.035″ wire, the arterial pressure measurements are performed (mean pressure needs to be lower than 15 mm Hg). In the next step, the CS is engaged with a multipurpose catheter; a standard 0.035″ wire is advanced distally into the CS, and venography is performed to evaluate the potential implantation area (adequate dimensions for device deployment—proximal diameter < 10 mm and >14 mm; absence of side branches or vascular anomalies). After switching to the 9 Fr guiding catheter, the CSR is implanted at 4–6 atmospheres with a device oversize of up to 20% relative to the CS size. A standard dose of unfractionated heparin (100 UI/kg) is administered during the procedure. Patients will receive additional dual antiplatelet therapy from the day of implantation for three months.

### 2.3. Follow-Up

All patients underwent an initial clinical evaluation by trained medical staff regarding past medical history. In addition, an in-depth analysis of angina symptoms was performed, including Canadian Cardiovascular Society (CCS) class assessment, Seattle Angina Questionnaire—7 items (SAQ-7) scores; New York Heart Association (NYHA) functional class, and 6-min walk test (6-MWT) along with transthoracic echocardiography (TTE). In addition, all patients were assessed for quality of life using the 36-item Short Form Health Survey (SF-36), the EQ-5D-5L questionnaire, and the EQ-VAS. At the end of the initial hospitalization, all clinical and procedural characteristics, including safety adverse events, were also collected. The primary time point for clinical re-evaluation (including all data collected during the initial evaluation) was set at 3 months.

### 2.4. Statistical Analysis

Data are presented as mean with standard deviation (SD) or median with interquartile range (IQR), depending on the normality of the distribution (assessed by Shapiro–Wilks test). Categorical data were analyzed using McNemar’s test. Continuous data were analyzed using Student’s paired *t*-test or Wilcoxon paired signed-rank test, depending on the results of the Shapiro–Wilks test for normality. McNemar’s test was used to compare changes in CCS levels. Post hoc comparisons of CCS subgroups were performed using McNemar’s test, comparing a given category with other categories, with the Holm–Bonferroni correction for multiple testing. Sample mean and 95% confidence interval for mean were used for the *t*-test, and sample pseudomedian and 95% confidence interval for pseudomedian were used for the Wilcoxon test. All tests were performed at a significance level of alpha = 0.05. The R statistical package was used for all analyses.

## 3. Results

The study includes a retrospective analysis of the mid-term results (3-month follow-up) of 55 consecutive patients who underwent implantation of the Sinus Coronary Reducer device between April 2022 and January 2024 at the Department of Cardiology, Cooper Health Centre Lubin, Poland. Baseline clinical characteristics are summarized in Table 1. The vast majority of subjects enrolled in the study were men (87.3%) with a mean age of 73.1 ± 6.9 years.

We observed a high burden of cardiovascular risk factors in the study cohort—hypertension (100%), hyperlipidemia (89.1%), diabetes (68.2%), or prediabetes (9.1%). Nearly nine out of ten patients underwent revascularization by PCI (85.9%), and more than half of the study population (58.2%) had previously undergone CABG surgery. Only four patients out of the study cohort had non-obstructive CAD (microvascular dysfunction); the rest were presented with obstructive CAD. Of these, the majority (62.7%) were considered to have previously undergone optimal revascularization, while the remaining were considered to be ineligible for further revascularization.

All patients included in the study had been on optimal pharmacological therapy for at least 3 months. The average number of anti-anginal medications used was 4 (3.5–5). Beta blockers were used by 94.5% of patients, long-acting nitrates 78.2%, trimetazidine 78.2%, and calcium channel blockers 54.5%.

The mean CCS angina class at baseline was 3.15 ± 0.6. A high angina burden was reported in all patients. It was highlighted by relatively low scores on the SAQ-7 total (39.2 ± 15.8) and poor exercise tolerance with a mean 6-min walk test of 233.3 ± 107.1 m. Poor angina control also affected baseline quality of life as measured by EQ-5D-5L (12.9 ± 3.9), SF-36 (112.7 ± 24.7), and EQ-VAS (51 ± 13.5).

Three months after implantation 22 (40%), subjects noticed an improvement in symptom control at one CCS class. Furthermore, 23 (41.8%) patients after reducer implantation demonstrated a 2 CCS class reduction, and 5 (9.1%) patients reported a 3 CCS class reduction (see Table 2). These favorable outcomes were confirmed by significant improvement in angina control, which was measured by SAQ-7 total (39.2 ± 15.8 vs. 50.4 ± 20.5; *p* < 0.0001), along with an increased abolition of physical limitation—6-MWT (233.3 ± 107.1 vs. 305.2 ± 126.8; *p* < 0.0001). In addition, patients reported a statistically significant improvement in quality of life 3 months after CSR implantation; all data on these clinical characteristics are summarized in Table 3. Anti-anginal pharmacotherapy remained unchanged during the observation period. In terms of safety outcomes, all patients underwent successful device deployment with no access site complications. In one case, we observed migration of the Coronary Sinus Reducer into the pulmonary arteries. In this particular case, we were able to retrieve the lost device using percutaneous loops. After successful extraction of a device during the same procedure, the patient underwent a second successful attempt at CSR implantation.

## 4. Discussion

In the presented single-center observational study, we observed good safety and efficiency of Coronary Sinus Reducer in a “real-life” population of patients with chronic disabling refractory angina pectoris without a revascularization option. There are three main findings of the study: (1) CS reducer implantation was a relatively safe procedure without serious adverse events requiring bailout cardiac surgery; (2) the clinical efficacy of this therapy was documented in real life by reducing disabling angina symptoms, improving quality of life and exercise tolerance; (3) despite variability in preimplant clinical presentation with concomitant limited support of advanced imaging (MRI/PET) during the qualification process, favorable clinical and safety results were maintained at mid-term.

Despite undeniable improvements in revascularization techniques and the pharmacological management of coronary artery disease, clinicians are often faced with patients presenting with disabling chronic refractory angina, although all therapeutic options have been applied in the treatment protocol. Paradoxically, as revascularization techniques and armamentarium evolve, the number of patients with severe refractory angina and a predictable long life expectancy is steadily increasing [8,9], and there is an urgent clinical need for novel therapies to alleviate symptoms and improve the quality of life in this group of patients.

Evidence for the safety and efficacy of SCR in non-optional patients with refractory angina pectoris is still accumulating, but the vast majority of it comes from a small number of observational registries or few randomized trials [10,11,12,13,14,15,16,17,18,19], which is reflected in the relatively low level of recommendation in revascularization guidelines [20].

The largest available multicenter study is the Reduce Study [21], which reported high procedural safety, effective implantation, and demonstrated a reduction in anginal symptoms (CCS class), improvement in the SAQ questionnaire, and a reduction in the average number of anti-anginal drugs.

Recent reports demonstrate the effectiveness of the coronary sinus reducer in reducing anginal symptoms, ref. [22] although they do not show significant improvement in perfusion in ischemic segments on cardiac magnetic resonance imaging. However, it should be noted that there are reports indicating a significant impact of the coronary sinus reducer on perfusion [23].

In our experience, all attempts at device implantation have ultimately resulted in successful CSR implantation. This high percentage of success is still slightly higher than the average results observed to date but seems to be in line with previously reported results [17]. This finding is probably related to an advanced proctoring program applied in our cardiac center along with the fact that the implantation procedure is always performed by two operators, one of whom has extensive experience in CRT-D implantation. Another added value is the intensive development of the CSR implant program at our site—we performed a relatively high number of procedures during a short training period. Furthermore, in our study cohort, we noticed only one periprocedural compilation (temporary dislocation of the device from coronary sinus), and such a low rate of complications (1,8%) is similar to other studies [19], suggesting a good safety profile. Moreover, from a practical standpoint, it is noteworthy that we routinely perform vascular access under ultrasound guidance, which allows us to eliminate access site complications despite the high burden of patients on anticoagulant therapy in the study cohort (30.9%).

It can be reasonably assumed that Coronary Sinus Implantation is a relatively safe procedure. A single, isolated periprocedural incident occurred in our study cohort: the Coronary Sinus Reducer migrated into the pulmonary arteries without a lasting effect on the patient’s prognosis. The lost device was successfully retrieved using percutaneous loops, and after the retrieval, the patient underwent a second successful attempt at CSR implantation during the same procedure. This specific case was subjected to a comprehensive revision by our team with the invaluable assistance of a proctor’s evaluation. It is presumed that during the initial attempt at implanting the device, the reducer was deployed in an inappropriate manner, resulting in its proximal edge being inadequately seated. This was likely due to the reducer being positioned too closely to the ostium of the coronary sinus. It was noteworthy that during the subsequent implantation attempt, we selected a more distal position and achieved an optimal outcome.

In our study cohort, despite the fact that qualification for the procedure was based mainly on clinical evaluation without the use of sophisticated imaging methods (MRI or PET), implantation of the reducer resulted in favorable results: the majority of patients 50 (90.9%) obtained results, which seems to be in line with a previously reported response rate, which was 71–85%. Three months after implantation, the average angina reduction reached a mean 1.6 ± 0.8 CCS class. These favorable results were confirmed by a significant improvement in angina control as measured by the SAQ-7 total questionnaire. Furthermore, these data were further objectified by an increase in physical activity capacity: 6-MWT distance increased significantly (233.3 ± 107.1 vs. 305.2 ± 126.8; *p* < 0.0001).

The clinical benefit achieved in the significant reduction in refractory angina was reflected in an improvement in the overall quality of life by all of the assessment methods used—EQ-5D-5L, SF-36 and EQ-VAS. These favorable results have potentially important practical implications; given that patients with uncontrolled refractory angina are more likely to suffer from depression and anxiety disorders [24,25], CSR may be a key to addressing these unmet clinical needs in this subpopulation. Furthermore, over time, it may help reduce economic costs and the burden on the healthcare system by reducing the number of unnecessary hospitalizations (our study cohort had an average of up to four hospitalizations due to refractory angina during the year prior to implantation).

### Limitations

Our study has several limitations. The main limitations of our study appear to be the lack of a control group and the relatively small number of patients enrolled. Another important shortcoming of our study is the lack of objective measurement of myocardial ischemia reduction after reducer implantation. Despite these limitations, this study appears to be valuable from a practical standpoint, since it includes data from one of the largest numbers of patients treated in a single-center, real-world setting.

## 5. Conclusions

Data from our real-world, single-center registry appear to confirm that CS reducer implantation is a safe and effective therapeutic option for patients with refractory angina who are not candidates for revascularization. CRS appears to be particularly effective in alleviating angina symptoms and improving quality of life. Further large, randomized trials with a control group are needed to fully investigate the impact of CRS on long-term clinical outcomes in this patient subpopulation.

## Figures and Tables

**Table 1:** Baseline characteristics.

Baseline Characteristics (n = 55)	
Age	73.1 ± 6.9
Male	48 (87.3%)
BMI (kg/m^2^)	28.6 ± 4.5
Hypertension	55 (100%)
Dyslipidemia	49 (89.1%)
Diabetes mellitus	32 (58.2%)
• Insulin-dependent	5 (9.1%)
Impaired glucose tolerance	5 (9.1%)
Nicotine addiction	21 (38.2%)
Coronary artery disease	55 (100%)
Years with CAD diagnosis (Q1–Q3)	14 (10–20)
Ejection fraction (%) (Q1–Q3)	55 (45–60)
Heart failure	13 (23.6%)
• NYHA Class I	1 (1.8%)
• NYHA Class II	12 (21.8%)
COPD	8 (14.5%)
Atrial fibrillation	17 (30.9%)
• Paroxysmal	14 (25.5%)
• Permanent	3 (5.5%)
Cerebrovascular events: Stroke/TIA	6 (10.9%)
Chronic renal disorder	11 (20%)
• Stadium G2	1 (1.8%)
• Stadium G3	10 (18.2%)
Peripheral arterial disease	20 (36.4%)
Coronary status	
Obstructive CAD	51 (92.7%)
Non-obstructive CAD	4 (7.3%)
Microvascular dysfunction	4 (14.5%)
Optimal revascularization status	32 (58.2%)
Disqualified from revascularization	19 (34.5%)
Post-MI	32 (58.2%)
• STEMI	15 (27.3%)
• NSTEMI	19 (34.5%)
Post-PCI	47 (85.5%)
• PCI LM	10 (18.2%)
• PCI LAD	23 (41.8%)
• PCI Cx	28 (50.9%)
• PCI RCA	36 (65.5%)
• PCI of bypass graft	6 (10.9%)
Number of DES (Q1–Q3)	7 (5–9)
Post CABG	32 (58.2%)
LIMA-LAD	27 (49.1%)
Ao-LAD	4 (7.3%)
Ao-Dg	46 (83.6%)
Ao-OM	20 (36.4%)
Ao-RCA	22 (40%)

Abbreviations: BMI—body mass index; CAD—coronary artery disease; NYHA—New York Heart Association; COPD—chronic obstructive pulmonary disease; TIA—transient ischemic attack; MI—myocardial infraction; STEMI—ST-segment-elevation myocardial infarction; NSTEMI—non-ST-segment myocardial infraction; PCI—percutaneous coronary intervention; LM—left main artery; LAD—left anterior descending artery; Cx—circumflex artery; RCA—right coronary artery; DES—drug eluting stent; CABG—coronary arteries bypass grafting; Dg—diagonal artery; OM—obtuse marginal artery.

**Table 2:** Change in CCS Class.

Change in CCS Class						
Baseline		3-month follow up				
		I	II	III	IV	*p* < 0.01
0	I	0	0	0	0	
6 (10.9%)	II	5	1	0	0	
35 (63.6%)	III	18	14	3	0	
14 (25.5%)	IV	5	5	3	1	
		28 (50.1%)	20 (36.4%)	6 (10.1%)	1 (1.8%)	

Table cells colored red correspond to an increase in CCS grade, yellow cells correspond to no change in CCS grade, green cells correspond to a decrease in CCS grade. Abbreviations: CCS—Canadian Cardiovascular Society.

**Table 3:** Questionaries and 6-minutes’ walk test results.

Seattle Angina Questionnaire-7 Items (SAQ-7)			
	Baseline	3-month	*p*
SAQ-7 total	39.2 ± 15.8	50.4 ± 20.5	<0.0001
SAQ-7-PL	40.2 ± 18.9	48.9 ± 26	0.0043
SAQ-7-AF	48.9 ± 22.9	65.3 ± 26.9	0.0002
SAQ-7-QL	29.1 ± 17	47.3 ± 24.3	<0.0001
6-min walk test (6MWT)			
	Baseline	3-month	*p*
Distance (meters)	233.3 ± 107.1	305.2 ± 126.8	<0.0001
EQ-5D-5L			
	Baseline	3-month	*p*
Score	12.9 ± 3.9	11.5 ± 3.8	0.01
EQ-VAS			
	Baseline	3-month	*p*
Score	51 ± 13.5	59.7 ± 17.6	0.0007
SF-36 Questionnaire			
	Baseline	3-month	*p*
Score	112.7 ± 24.7	92.3 ± 28.3	<0.0001

Abbreviations: PL—physical limitations; AF—angina frequency; QL—quality of life; VAS—visual analog scale.

## Data Availability

All data not included in the manuscript are available after contacting the corresponding author in accordance with local legal regulations.
